# Agronomic Efficiency of *Bacillus* spp. Co-Inoculation Associated with Phosphorus Fertilization Levels in Soybean and Maize Cultivation

**DOI:** 10.3390/microorganisms14081620

**Published:** 2026-07-24

**Authors:** André Sarabia Zamarian, Ana Claudia Botelho, Ricardo Robson Trautmann, Tauane Santos Brito, Andre Gustavo Battistus

**Affiliations:** 1 Tecnomyl S.A., Av. Aviadores del Chaco n° 3301, Asunción 1110, Paraguay; tauane.brito@tecnomyl.com (T.S.B.); andre.battistus@tecnomyl.com (A.G.B.); 2 Tecnomyl Brasil, St. Professor Joao Argemiro Loyola, n° 319—Seminario, Curitiba 80240-530, Paraná, Brazil; ana.botelho@tecnomyl.com; 3 Agrofértil, Av. San Blas/Pablo Neruda—Km. 6,5 Ciudad del Este, Ciudad del Este 7000, Alto Paraná, Paraguay; ricardo.trautmann@agrofertil.com.py

**Keywords:** *Bacillus megaterium*, *Bacillus subtilis*, phosphate solubilization, microbial association, plant growth-promoting bacteria

## Abstract

The co-inoculation of *Bacillus megaterium* (CCT 2482) and *Bacillus subtilis* (CCT 3131) may improve plant phosphorus nutrition and agronomic performance under field conditions. This study evaluated the agronomic efficiency of co-inoculation via seed treatment, combined with different phosphate fertilizer doses, in the morphophysiological and productive performance of corn and soybean under distinct edaphoclimatic conditions. Eight trials (four per crop) were conducted during the 2023/2024 season in a randomized block design with eight treatments and four replications. In soybean, co-inoculation increased leaf phosphorus content, grain mass, number of pods per plant, thousand-grain weight, and yield, even with a 25% reduction in phosphate fertilization. In corn, it improved shoot and root biomass, grains per row, thousand-grain weight, and productivity. These results suggest that co-inoculation may contribute to phosphate fertilization management, although direct comparisons across fertilization levels indicate that its effect depends on crop, variables, and the environment. The bacterial strains exhibited strong potential for phosphate solubilization and plant growth promotion across varied environmental conditions, confirming their adaptability. Therefore, this approach may serve as a complementary strategy for phosphate fertilization management, contributing to improved agronomic performance and more sustainable agricultural production systems.

## 1. Introduction

Some bacteria have the ability to act directly in the solubilization of inorganic phosphates and in the mineralization of phosphorous organic compounds, assisting in the access of agricultural crops to the nutrient, as well as indirectly promoting plant growth and health, resulting in increased productivity [1]. Among the genera with this capacity, the best known and most widely used include *Pseudomonas*, *Enterobacter*, *Azospirillum*, *Clostridium*, *Azotobacter*, *Arthrobacter*, *Rhizobium* and *Bacillus* [2].

The genus *Bacillus* has stood out among the phosphate solubilizers for acting to promote plant development by direct mechanisms, such as the production of phytohormones and biocontrol action, and indirect mechanisms [3,4], such as increased resistance to abiotic and biotic stresses [5,6]. In addition, the development of the genus encompasses an endospore phase, which acts as a resistance structure, enabling the use of the microorganism in several areas of agriculture, including mixing with essential chemicals in management techniques [7].

Among the genera of *Bacillus*, the most widespread is *Bacillus subtilis*, which is widely used in the control of leaf and root diseases, and which also acts in the production of phytohormones and in nutritional cycling, in addition to inducing systemic resistance in plants [8]. *Bacillus megaterium* is a species with a high production capacity of organic acids, acting directly on the dynamics of phosphorus in the soil, releasing the nutrients adhered to the soil colloids and making them available to plants throughout the cycle, thus optimizing the use of phosphate fertilizers [7].

The co-inoculation of *B. subtilis* and *B. megaterium* seeks to combine strains that act in a complementary way with respect to the availability of phosphates, hormone production, siderophores, and biofilms, without antagonizing each other, or that act in combination with other beneficial microorganisms such as *Azospirillum* and *Bradyrhizobium* [8,9].

In experiments conducted under different soil and climatic conditions, the application of these bacteria via seed treatment resulted in greater absorption of phosphorus, increased root and shoot biomass, and increased grain productivity [10]. In maize, co-inoculation contributed to increased phosphorus use efficiency, promoting better initial seedling development and greater uniformity in the plant stand [11]. In soybeans, increases in photosynthetic rate and chlorophyll contents were observed, indicating that the presence of *Bacillus* may favor plant metabolism, contributing to productivity [12].

In view of the above, the present study sought to determine the agronomic efficiency of the co-inoculation of *Bacillus megaterium* (CCT 2482) and *Bacillus subtilis* (CCT 3131) via seed treatment, with different doses of phosphate fertilizer, in the morphophysiological and productive performance of corn and soybeans grown in different edaphoclimatic regions.

## 2. Materials and Methods

### 2.1. Characterization of Experimental Areas

The tests were conducted on soybean and corn crops in four edaphoclimatic regions (Artur Nogueira, São Paulo; Bandeirantes, Paraná; Ouro, Santa Catarina; and Ponta Grossa, Paraná).Eight tests were conducted during the 2023/2024 harvest, as detailed in Table 1, and fertility analyses of the areas in question are provided in Table 2.

**Figure 1:**
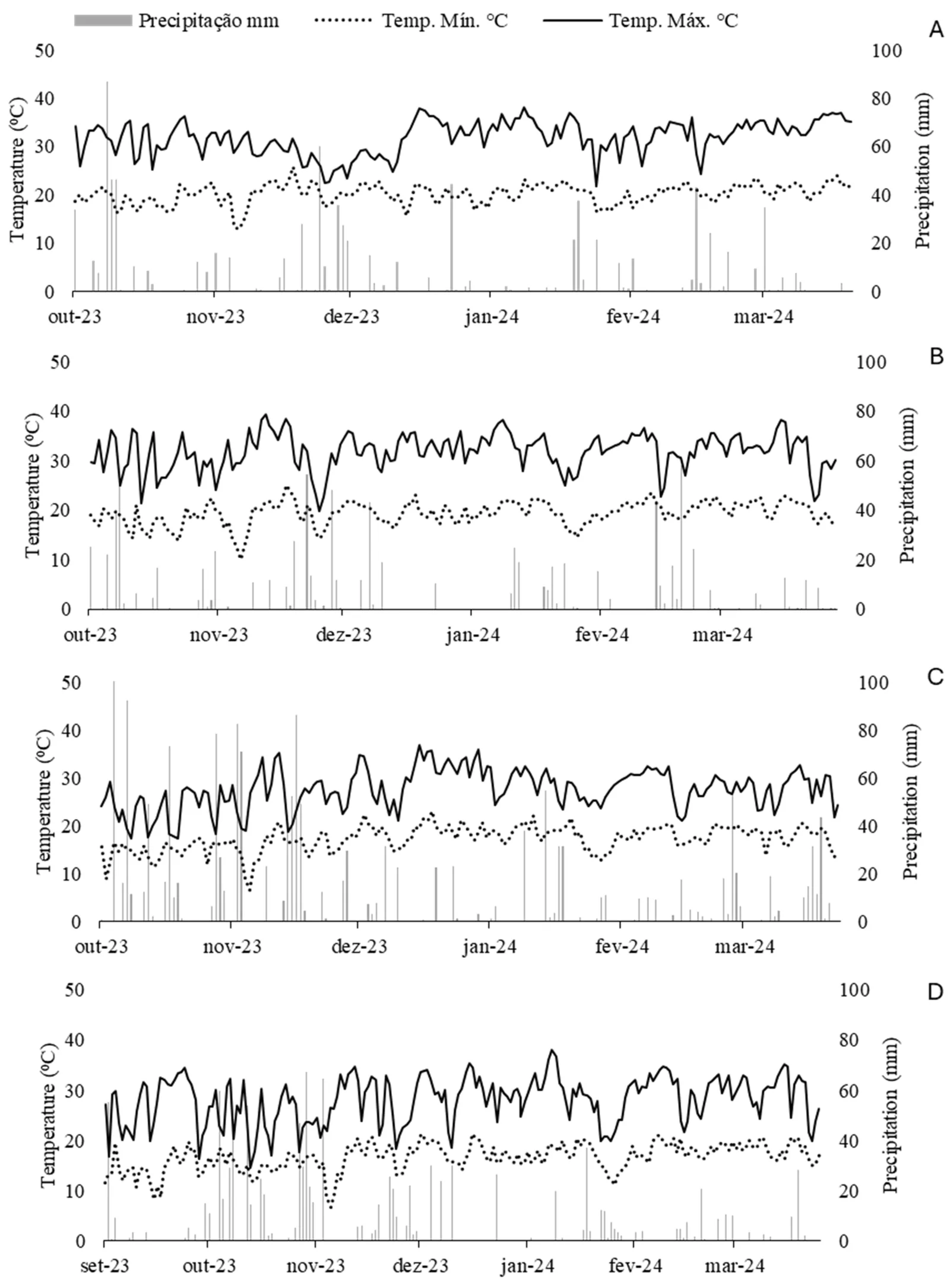
Monthly averages of average temperature (°C) and accumulated rainfall (mm) in the region of Artur Nogueira, SP (**A**); Bandeirantes, PR (**B**); Ouro, SC (**C**); and Ponta Grossa, PR (**D**), during the experimental period (2023/2024 harvest).

In the municipality of Artur Nogueira, São Paulo, the tests were implemented at latitude 22°31′52.53″ S and longitude 47°07′5.11″ W, with an altitude of 596 m. The soil is classified as Acriferric Red Latosol, with a clayey texture [13], and with a pH of 4.20. According to Köppen [14], the climate of the region is classified as tropical with dry winter (Aw), with an average temperature of 21.7 °C and average precipitation of 1484 mm (Figure 1).

In the municipality of Bandeirantes, Paraná, the tests were implemented at latitude 23°6′53.18″ S and longitude 50°21′22.74″ W, with an altitude of 449 m. The soil is classified as Eutroferric Red Latosol, with a very clayey texture [13], and with a pH of 5.73. According to Köppen [14], the climate of the region is classified as tropical (Aw), with an average temperature of 22.2 °C and average rainfall of 1339 mm (Figure 1).

In the municipality of Ouro, Santa Catarina, the tests were implemented at latitude 27°18′14.11″ S and longitude 51°37′56.29″ W, with an altitude of 526 m. The soil is classified as moderate Tb A Eutrophic Cambisol, with a clayey texture, and moderate A Eutrophic Litholics, with a medium texture, both stony and in a strongly wavy relief [13], with a pH of 4.71. According to Köppen [14], the climate of the region is classified as humid subtropical (Cfa), with an average temperature of 22 °C and average rainfall of 1210 mm (Figure 1).

In the municipality of Ponta Grossa, Paraná, the tests were implemented at latitude 25°9′53.85″ S and longitude 50°11′23.79″ W, with an altitude of 799 m. The soil is classified as typical Tb Eutrophic Haplic Cambisol, with a clayey texture [13], and with a pH of 5.35. According to Köppen [14], the climate of the region is classified as temperate humid (Cfb), with an average temperature of 18.9° C and average precipitation of 1371 mm (Figure 1).

### 2.2. Climate Data

Climatic data throughout the study in each location were collected at a local weather station from the first date of the month of planting to the date of harvest.

### 2.3. Experimental Design and Treatments

The trials followed a randomized block design with eight treatments and four replications. The treatments are detailed in Table 3. The experimental plots were 4 m wide by 6 m long, with a total area of 24 m^2^.

The commercial product was used as a market reference treatment. Its identity is not disclosed due to commercial confidentiality; however, its declared concentration was 4.4 × 10^9^ CFU mL^−1^, whereas the tested product contained 2.0 × 10^8^ CFU mL^−1^. This difference was considered when interpreting the results and is acknowledged as a limitation of the study.

For soybean, the variety HO Pirapó IPR (HO Genética, Goiânia, Brazil) was used, for corn, the hybrid Feroz Vip 3 (Syngenta, São Paulo, Brazil). All inoculants were applied via seed treatment according to the doses indicated in Table 3. Inoculation was performed manually on 1000 g of seeds.

Treatments with phosphorus application were implanted at the time of sowing, using formulation 00-28-00 NPK as a source. The phytosanitary treatments were carried out according to the evolution of the crops throughout the harvest.

### 2.4. Evaluations

#### 2.4.1. Phytotoxicity

At seven days after emergence, the phytotoxic effect of the test product *Bacillus megaterium* CCT 2482 + *Bacillus subtilis* CCT 3131—2.0 − 10^8^ CFU mL^−1^ was evaluated using the grade scale [15,16].

#### 2.4.2. Morphometric, Nutritional and Production Analyses

##### Soybean

At 35 days after emergence (35DAE) and at the phenological stages V4, flowering, and R3, 5 complete plants were evaluated in the central area of each plot, accounting for the number of nodules per plant and the dry mass of the shoot and roots.

To evaluate the leaf content of nutrients (nitrogen, phosphorus and potassium), a sample composed of indicator leaves was taken at the R1 stage. Leaf nitrogen was determined by the semi-micro Kjeldahl method, phosphorus by spectrometry, and potassium by emission flame spectrometry [17].

The productivity was evaluated at 7.5 m^2^ per plot, together with the thousand-seed weight (TSW), the number of pods per plant and the number of grains per pod and plant population. Additionally, a sample of the grains per treatment was evaluated to determine the accumulation of nutrients in the grains, using the same methodology for leaf analysis.

##### Corn

At the phenological stages V4, flowering, and R3, 5 complete plants were collected in the central area of each plot to determine the dry mass of the shoot and roots. For the evaluation of the leaf contents of nitrogen, phosphorus and potassium, a sample composed of indicator leaves was taken below the ear for corn at the VT stage and analyzed according to the methodologies mentioned for soybean [17].

The productivity was evaluated at 3.0 m^2^ per plot, together with the thousand-seed weight (TSW), the number of ears per plant, the number of rows of grains per ear and the number of grains per row. Additionally, a sample of the grains per treatment was evaluated to determine the accumulation of nutrients in the grains, according to the methodology mentioned above.

### 2.5. Statistical Analysis

The data obtained were submitted to normality and homoscedasticity analysis, followed by analysis of variance (ANOVA) for each crop. When there was a significant difference at 5% probability of error, the means of the treatments were compared by Dunnet’s test at the level of 95% significance.

A joint analysis across locations was performed for each crop, considering treatments as fixed effects and environments as random effects. The treatment × environment interaction was evaluated to assess the stability and consistency of treatment responses across environments. When significant effects were detected, treatment means were compared using Tukey’s test at 5% probability. Dunnett’s test was additionally used to compare treatments with the uninoculated and unfertilized control.

## 3. Results

### 3.1. Phytotoxic Effect and Physiological Potential of Seeds

No visible phytotoxicity symptoms were observed seven days after emergence in either soybean or maize, including chlorosis, necrosis, deformation, delayed emergence, or reduction in early seedling vigor.

### 3.2. Soybean

The co-inoculation of *Bacillus megaterium* (CCT 2482) and *Bacillus subtilis* (CCT 3131) with 75% of the phosphate fertilization dose resulted in a significant increase in area dry mass compared to the control treatment in soybean crop, flowering, and grain filling, resulting in higher averages than the isolated use of complete phosphate fertilization and the combination of complete fertilization with the evaluated *Bacillus* (Table 4).

Inoculation with commercial products resulted in increased nitrogen accumulation in the grains in co-inoculated and co-inoculated plants fertilized with 100% of the phosphate fertilization dose (Table 5). Phosphorus accumulation in leaves was higher in the co-inoculated plants that received 50 and 75% of the fertilization, as well as in grains at all doses of phosphate fertilization combined with *Bacillus megaterium* (CCT 2482) and *Bacillus subtilis* (CCT 3131). Potassium accumulation was statistically higher with the use of bacterial co-inoculation, with little dependence on the dose of phosphate fertilization used.

The number of pods was statistically higher in co-inoculation plants of *B. megaterium* (CCT 2482) and *B. subtilis* (CCT 3131) associated with 75% and 100% phosphate fertilization and with the isolated commercial product (Table 6). All treatments significantly increased the thousand-seed weight (TSW) compared to the non-co-inoculated and non-fertilized control (Table 6).

Productivity was higher in plants co-inoculated with *Bacillus megaterium* (CCT 2482) and *Bacillus subtilis* (CCT 3131) at all fertilization doses evaluated, in addition to isolated phosphate fertilization and the combination of the commercial product with the complete dose of phosphate fertilization (Table 6).

### 3.3. Corn

Co-inoculated *Bacillus megaterium* (CCT 2482) and *B. subtilis* (CCT 3131), combined with 50, 75 and 100% of the phosphate fertilization, increased the accumulation of shoot and root mass at the different evaluation times compared to the uninoculated and unfertilized control (Table 7).

Co-inoculation with *B. megaterium* (CCT 2482) and *B. subtilis* (CCT 3131), without phosphate fertilization and with 50 and 75% of the phosphate fertilization, significantly increased the number of grains per row compared to the uninoculated control (Table 8). The thousand-seed weight (TSW) was higher in all co-inoculation treatments of *B. megaterium* (CCT 2482) and *B. subtilis* (CCT 3131) (with or without phosphate fertilization), as was the productivity of plants treated with a co-inoculation of *B. megaterium* (CCT 2482) and *B. subtilis* (CCT 3131), or with the commercial product combined with 100% phosphate fertilization, compared to the uninoculated and untreated control (Table 8).

## 4. Discussion

Unlike nitrogen, phosphorus uptake is highly dependent on the pH of the soil in which cultivation was carried out [18]. The absorption of this nutrient is slow via diffusion [19]; thus, the use of inoculation with phosphorus-solubilizing microorganisms can act during the process, stimulating the root development of the plant concomitant with the production of organic acids, which act in soil acidification so that P_2_O_5_ is converted into H_2_PO_4_ or HPO_4_^2−^ [18].

In this study, evaluating the initial soil conditions in the test regions, it is possible to notice two distinct situations, in addition to the particularities of each of the experimental stations. In two locations, there is acidic soil with a low phosphorus concentration, and in two other locations, there is acidic soil and a medium-high phosphorus concentration. However, in one of the high-concentration sites, the high acidity affects plant development and the action of microorganisms, mainly due to the high levels of aluminum (Artur Nogueira, SP) (Table 2), reducing the potential effects of inoculation on the plants evaluated.

In grain filling, little phosphorus comes from root absorption; phosphorus is easily redistributed via phloem and is sent to the grain in formation via plant remobilization [20]. These results suggest that early plant–microorganism interactions may contribute to nutrient accumulation during vegetative development, which can subsequently support grain filling.

In the present study, treatments with greater shoot dry matter accumulation and higher foliar phosphorus concentrations were also associated with higher phosphorus accumulation in grains, suggesting nutrient remobilization during grain filling (Table 4 and Table 5).

Potassium is highly dependent on the water to be absorbed [8], and in leaves, in addition to enzymatic activation, it is an essential nutrient in stomatal opening, acting in the substrate entry process as part of the photosynthetic process, and, as it is not a structural nutrient, it can be easily remobilized from leaves to grains when the filling phase begins [20]. The greater root biomass observed in inoculated plants may be associated with physiological mechanisms previously reported for *Bacillus* spp., including effects on root growth and nutrient acquisition [8,21]. Increased root development may favor soil exploration and resource uptake, contributing to the agronomic responses observed in both crops (Table 4, Table 5, Table 6, Table 7 and Table 8).

Previous studies evaluating the co-inoculation of *B. subtilis* (B119) and *B. megaterium* (B2084) in association with phosphate fertilization have reported increases in soybean productivity, particularly under conditions of lower soil fertility [8,22]. Similar agronomic responses were observed in the present study (Table 6).

In studies conducted by Guimarães et al. [21], doses of inoculant containing *Bacillus megaterium* (B119) and *Bacillus subtilis* (B2084) (via seed or furrow), combined with 50% of the phosphate fertilization, resulted in a level of soybean and corn productivity similar to that in plants grown with 100% of the phosphate fertilization (triple superphosphate) without co-inoculation, indicating the significance of bacterial action in promoting plant growth and optimizing available phosphorus, as corroborated by the present study.

Previous studies have reported positive effects of *Bacillus* strains on maize productivity under contrasting phosphorus availability conditions. Under low-phosphorus fertility, inoculation with B119 increased grain phosphorus content, while B116 promoted significant yield gains compared to uninoculated controls [21]. These findings are consistent with the positive agronomic responses observed in the present study.

The ecological characteristics of *Bacillus* strains confer additional advantages for agricultural systems. Their ability to form endospores and tolerate adverse environmental conditions, such as drought and high temperatures, contributes to their adaptability under field conditions [23,24]. In the present study, co-inoculation with CCT 2482 and CCT 3131 was associated with increased vegetative growth and improved agronomic performance in both crops, reinforcing the potential of *Bacillus*-based inoculation as a complementary strategy within phosphate fertilization management programs.

## 5. Conclusions

The co-inoculation of *Bacillus megaterium* (CCT 2482) and *Bacillus subtilis* (CCT 3131) via seed treatment promotes corn and soybean development, generating gains in vegetative growth and productivity, especially in association with reduced doses of phosphate fertilizer. The action of co-inoculation was particularly important in the early stages of the cycle due to the accumulation of phosphorus, which was subsequently redistributed to the grains during the filling process.

The strains used (CCT 2482 and CCT 3131) were associated with improved plant growth and agronomic performance under different experimental conditions, reinforcing their applicability in diverse agricultural environments. Co-inoculation of *Bacillus* strains may serve as a complementary strategy in phosphate fertilization programs, contributing to sustainable crop production under a range of environmental conditions.

## Figures and Tables

**Table 1:** Culture, location, sowing period and harvest of the crops planted under doses of phosphate fertilization and/or co-inoculation with *Bacillus megaterium* + *Bacillus subtilis* in the 2023/2024 harvest in different regions.

Crop	Location	Sowing	Harvest
Soybean	Artur Nogueira (SP)	23 October 2023	29 February 2024
	Bandeirantes (PR)	18 October 2023	20 March 2024
	Ouro (SC)	25 October 2023	23 March 2024
	Ponta Grossa (PR)	10 October 2023	14 March 2024
Corn	Artur Nogueira (SP)	23 October 2023	20 March 2024
	Bandeirantes (PR)	18 October 2023	28 March 2024
	Ouro (SC)	25 October 2023	5 March 2024
	Ponta Grossa (PR)	11 October 2023	6 March 2024

**Table 2:** Physicochemical analyses carried out at a depth of 0 to 20 cm in the four experimental regions, prior to the implementation of soybean and corn crops in the 2023/2024 harvest.

Area	pH ^1^	H + Al	Al	Ca	Mg	K	SB	CEC	P ^2^	MO	V	Clay	Silt	Sand
		mmolc_dm3-1_							mg^dm3-1^	g^dm3-1^	%			
Arthur Nogueira—SP	4.20	72.56	6.05	19.80	2.60	3.09	25.49	98.05	21.80	18.40	26.00	46.45	12.25	41.30
Bandeirantes—PR	5.73	31.94	0.00	37.80	19.90	6.40	64.10	94.06	16.10	42.34	66.74	44.75	18.10	37.15
Ouro—SC	4.71	52.81	2.47	51.70	15.70	8.27	75.67	128.48	70.00	41.93	58.90	55.55	37.60	6.85
Ponta Grossa—PR	5.35	90.47	9.86	28.40	5.80	2.90	37.10	127.57	20.50	89.39	29.08	-	-	-

^1^ pH in water. ^2^ Analysis by Mehlich methodology.

**Table 3:** Description of the treatments applied in both crops in order to evaluate the effectiveness of the test product in promoting growth.

Treatments	Inoculant		Fertilizer Dose (kg ha^−1^)	Inoculant Dose (mL ha^−1^)
	Active Ingredient	Concentration (CFU mL^−1^)		
Control	-	-	-	-
Phosphate fertilization 100% (P *)	-	-	100	-
Commercial product	*Bacillus megaterium* BRM 119 *Bacillus subtilis* BRM 2084	4.4 × 10 ^9^	-	100
Commercial product + 100% P	*Bacillus megaterium* BRM 119 *Bacillus subtilis* BRM 2084	4.4 × 10 ^9^	100	100
*Bacillus*	*Bacillus megaterium* CCT 2482 *Bacillus subtilis* CCT 3131	2.0 × 10^8^	-	100
*Bacillus* + 50% P	*Bacillus megaterium* CCT 2482 *Bacillus subtilis* CCT 3131	2.0 × 10^8^	50	100
*Bacillus* + 75% P	*Bacillus megaterium* CCT 2482 *Bacillus subtilis* CCT 3131	2.0 × 10^8^	75	100
*Bacillus* + 100% P	*Bacillus megaterium* CCT 2482 *Bacillus subtilis* CCT 3131	2.0 × 10^8^	100	100

* P—phosphorus; CFU—colony forming unit.

**Table 4:** Shoot and root dry mass of soybean at different phenological stages.

Treatments	Dry Mass					
	Aerial Part			Roots		
	V4	Flowering	R3	V4	Flowering	R3
Control	2.63	16.83	26.14	1.13	2.59	3.92
Phosphate fertilization 100%	2.77	18.73 *	30.32 *	1.20	2.80	4.26
Commercial product	2.81	18.48	29.40 *	1.39	2.89	4.28
Commercial product + P 100%	2.88	18.45	29.62 *	1.25	2.95	4.52
*B. megaterium* + *B. subtilis*	2.98	16.72	25.85	1.18	2.83	4.18
*B. megaterium* + *B. subtilis* + 50% P	3.02	18.09	27.96	1.39	3.04	4.37
*B. megaterium* + *B. subtilis* + 75% P	3.12	20.13 *	32.62 *	1.27	2.97	4.43
*B. megaterium* + *B. subtilis* + 100% P	3.13	18.23	27.59	1.15	2.87	4.26
**Average**	2.92	18.21	28.69	1.25	2.87	4.28
**C.V. (%)**	10.09	6.32	6.30	23.57	9.18	4.83

*—Statistical difference by Dunnett’s test at 5% probability of error. P—Phosphorus.

**Table 5:** Joint analysis of nitrogen, phosphorus and potassium for R1 stages and soybeans under co-inoculation with *Bacillus megaterium* (CCT 2482) and *Bacillus subtilis* (CCT 3131) and/or phosphate fertilization doses in the 2023/2024 harvest in different edaphoclimatic regions.

Treatments	Nitrogen (g kg MS^−1^)		Phosphorus (g kg MS^−1^)		Potassium (g kg MS^−1^)	
	Leaf	Grain	Leaf	Grain	Leaf	Grain
Control	29.93	23.00	4.87	5.35	12.23	8.51
Phosphate fertilization 100%	32.76	24.62	5.81	5.81	14.30 *	9.81
Commercial product	31.19	26.73 *	6.15	6.28	15.02 *	9.20
Commercial product + P 100%	31.82	26.30 *	6.22 *	6.22	14.47 *	8.82
*B. megaterium* + *B. subtilis*	28.41	21.92	6.27 *	6.35	16.10 *	8.22
*B. megaterium* + *B. subtilis* + 50% P	31.07	24.70	6.09	6.83 *	14.07 *	10.33 *
*B. megaterium* + *B. subtilis* + 75% P	31.01	23.76	6.19 *	6.72 *	13.88	10.12 *
*B. megaterium* + *B. subtilis* + 100% P	31.39	23.85	6.18	6.72 *	13.86	8.89
**Average**	30.95	24.36	5.97	6.28	14.24	9.24
**C.V. (%)**	7.88	6.76	7.65	8.54	8.23	8.28

*—Statistical difference by Dunnett’s test at 5% probability of error. P—Phosphorus.

**Table 6:** Joint analysis of soybean production components under co-inoculation with *Bacillus megaterium* (CCT 2482) and *Bacillus subtilis* (CCT 3131) and/or phosphate fertilization doses in the 2023/2024 harvest in different edaphoclimatic regions.

Treatments	No. of Pods per Plant ^−1^	TSW (g)	Productivity (kg ha^−1^)
Control	49.72	167.04	3919.08
Phosphate fertilization 100%	56.61 *	176.33 *	4154.22 *
Commercial product	56.93 *	178.52 *	4043.62
Commercial product + P 100%	54.03	174.93 *	4120.64 *
*B. megaterium* + *B. subtilis*	51.59	183.21 *	3953.22
*B. megaterium* + *B. subtilis* + 50% P	54.56	176.52 *	4132.65 *
*B. megaterium* + *B. subtilis* + 75% P	60.17 *	173.88 *	4133.91 *
*B. megaterium* + *B. subtilis* + 100% P	55.16 *	173.93 *	4176.39 *
**Average**	54.85	175.55	4079.22
**C.V. (%)**	7.63	3.15	2.97

*—Statistical difference by Dunnett’s test at 5% probability of error. P—Phosphorus; TSW—thousand-seed weight.

**Table 7:** Joint analysis of shoot and root dry mass for stages V4, R1 and R3 of maize under co-inoculation with *Bacillus megaterium* (CCT 2482) and *Bacillus subtilis* (CCT 3131) and/or doses of phosphate fertilization in the 2023/2024 harvest in different edaphoclimatic regions.

Treatments	Dry Mass					
	Aerial Part			Roots		
	V4	Flowering	R3	V4	Flowering	R3
Control	55.51	413.64	467.49	13.09	301.42	324.70
Phosphate fertilization 100%	58.09	453.36	530.74 *	14.68	334.48	358.81
Commercial product	57.41	431.34	487.50	14.68	321.97	359.19
Commercial product + P 100%	57.54	477.38 *	511.24	14.86 *	341.00	364.00
*B. megaterium* + *B. subtilis*	57.93	477.07 *	504.64	14.23	342.92	372.50
*B. megaterium* + *B. subtilis* + 50% P	58.01	467.40 *	520.63	14.39	369.84 *	409.63 *
*B. megaterium* + *B. subtilis* + 75% P	58.30 *	468.13 *	532.59 *	14.70 *	364.31 *	441.25 *
*B. megaterium* + *B. subtilis* + 100% P	58.79 *	483.55 *	528.75 *	14.38	387.10 *	433.94 *
**Average**	57.70	458.98	510.45	14.38	345.38	383.00
**C.V. (%)**	3.60%	9.55%	10.02%	6.18%	13.64%	12.73%

*—Statistical difference by Dunnett’s test at 5% probability of error. P—Phosphorus.

**Table 8:** Joint analysis of corn production components under co-inoculation with *Bacillus megaterium* (CCT 2482) and *Bacillus subtilis* (CCT 3131) and/or phosphate fertilization doses in the 2023/2024 harvest in different edaphoclimatic regions.

Treatments	No. of Grains per row^−1^	TSW (g)	Productivity (kg ha^−1^)
Control	34.81	259.13	8514.10
Phosphate fertilization 100%	36.27	269.35 *	8861.34
Commercial product	36.51	268.12	8856.40
Commercial product + P 100%	36.73	270.70 *	8954.30 *
*B. megaterium* + *B. subtilis*	40.16 *	273.26 *	8702.83
*B. megaterium* + *B. subtilis* + 50% P	38.45 *	272.34 *	8877.83
*B. megaterium* + *B. subtilis* + 75% P	38.26 *	268.80 *	8824.33
*B. megaterium* + *B. subtilis* + 100% P	36.43	273.30 *	9042.12 *
**Average**	37.20	269.37	8829.16
**C.V. (%)**	7.11%	2.89%	4.02%

*—Statistical difference by Dunnett’s test at 5% probability of error. P—Phosphorus; TSW—thousand-seed weight.

## Data Availability

The data supporting the findings of this study are available from the corresponding author upon reasonable request.
